# Optimisation of a functional mycobacterial growth-inhibition assay to improve its suitability for infant TB vaccine studies^☆^

**DOI:** 10.1016/j.jim.2013.05.006

**Published:** 2013-08-30

**Authors:** S. Burl, B.S. Holder, B.K.M. Lo, B. Kampmann

**Affiliations:** a Department of Paediatrics, St Mary's Campus, Imperial College London, Norfolk Place, London W2 1PG, UK; b MRC Unit, The Gambia, Atlantic Road, Fajara, Gambia

**Keywords:** BCG-*lux*, *Mycobacterium tuberculosis*, Paediatric tuberculosis, Vaccine immunogenicity

## Abstract

The development of vaccines against tuberculosis continues to be hindered by the lack of correlates of protection. Immunity to *Mycobacterium tuberculosis* (*M.tb)* infection relies predominantly on cell mediated response, which is routinely measured using a read-out of host cytokine profiles. However, to date none of the cytokine profiles have been found to predict protection. A number of functional *in vitro* approaches that measure growth of mycobacteria pre- and post-vaccination as a potential functional surrogate marker for vaccine take have been developed. The use of a reporter-gene tagged BCG-*lux* assay measuring the viability of mycobacteria in whole blood samples has previously been described by our group to assess vaccine immunogenicity. Since only very small blood samples are usually available in paediatric studies, we now report a modification of the BCG-*lux* assay to reduce the volume required and make it more field-friendly. Our results show that a 2-fold reduction in blood volume made no significant difference to bacterial growth ratios, used as the main read-out. These results confirm the suitability of the BCG-*lux* assay for functional studies of vaccine immunogenicity and immunopathogenesis in young children and could play a role in late-phase TB vaccine trials of novel candidates.

## Highlights

• A 2-fold reduction in blood volume can now be used in a functional BCG-*lux* assay.
• This growth-restriction assay is a useful tool for vaccine assessment.
• Reduction in blood volumes will aid its use in trials of TB vaccines in infants.

## 1 Introduction

A major obstacle to the development of new vaccines against tuberculosis (TB) is the absence of an appropriate *in vitro* correlate to predict efficacy of a vaccine. Such a correlate would significantly reduce the need for expensive and extensive phase 3 trials. In addition to clinical disease endpoints, trials of vaccines conventionally measure antibody titres in response to the given vaccine, but this approach is not suitable to assess vaccines against TB, since cell-mediated responses rather than antibody are known to be the key mediators of protection. The immune response to *Mycobacterium bovis* bacillus Calmette–Guérin (BCG) vaccination (at present the only licensed vaccine against tuberculosis) has traditionally been monitored by the tuberculin skin test, measuring delayed-type hypersensitivity (DTH) to intradermal inoculation of purified protein derivative (PPD), a crude mixture of antigenic proteins from *M.tb*. Tuberculin sensitivity is also induced by exposure to *M.tb* itself, and some non-tuberculous mycobacteria, which interferes with the specificity of the TST. In addition conversion to PPD-positivity does not necessarily correlate with induction of protective immunity (Fine et al., 1994). More recently, production of gamma interferon (IFNγ) by peripheral blood T cells stimulated by mycobacterial antigens *in vitro* (*i.e.* the interferon gamma release assays (IGRA)) has also been used as a measure of exposure to *M.tb* infection or vaccine-take (Pai et al., 2008), but while there is extensive evidence that IFNγ-secreting T cells are an essential component of immunity, there is poor evidence that the ability of a vaccine to prime such cells is correlated with its protective efficacy (Kagina et al., 2010). Although it is likely that many other cytokines, in addition to IFNγ, are involved in the protection, the holy grail of the “correlate(s) of protection” against tuberculosis still remains to be found.

The present lack of suitable correlates of human protection has encouraged the development of *in vitro* models that incorporate possible mechanisms of growth restriction or mycobacterial killing as a functional read-out. Two *in vitro* methods of studying mycobacterial growth using isolated peripheral blood mononuclear cells have been developed and employed in BCG and *M.tb* growth restriction assays (Silver et al., 1998; Worku and Hoft, 2000). One method involves unstimulated lymphocytes in the primary lymphocyte assay (Silver et al., 1998) and the other uses antigen stimulated lymphocytes for the detection of memory immunity (Worku and Hoft, 2000). More recently, Kampmann et al. developed a whole blood assay to study mycobacterial survival, using a luciferase reporter system (Kampmann et al., 2000). This assay utilises vectors that contain a modified version of either BCG (rBCG-*lux*) or *M.tb* (rMtb-*lux*). The modification includes addition of a reporter enzyme (luciferase lux gene) that luminesces after the addition of an appropriate external substrate. Measurement of this signal directly relates to the numbers and viability of the mycobacteria (Kampmann et al., 2000). Although all of these assays have shown the capability of detecting increased mycobacterial growth inhibition after BCG vaccination, the whole blood assay is simpler to perform without the need for isolation of PBMCs and requires less blood making it an attractive assay for the field and for paediatric studies.

A number of studies have already been published which have used this assay to dissect the mechanisms that restrict or promote mycobacterial growth. In a proof-of principle study, immunogenicity of the BCG vaccine in infants has been demonstrated through greater control of BCG growth in the BCG-*lux* assay following BCG-vaccination of infants (Kampmann et al., 2004). Other studies showed that vitamin D played an important role in restriction of BCG and *M.tb* growth, dependent on neutrophils and in particular anti-microbial peptides (Martineau et al., 2007). A clinical trial of 2.5 mg of vitamin D supplementation (ergocalciferol) in TB contacts supported this finding. Studies in HIV positive children showed that prior to HIV anti-retroviral treatment (HAART) those with low CD4 T cell counts showed limited ability to restrict the growth of mycobacteria, but that the introduction of HAART led to rapid and sustained reconstitution of anti-mycobacterial immune responses, measured as the decreased growth of mycobacteria compared to HAART-naïve baseline (Kampmann et al., 2006).

At present a minimum of 4 mL of blood is used for these assays, which is often the maximum volume that can be collected from a young infant. Since it is likely that early anti-TB vaccine trials would wish to analyse vaccine responses in more than one assay system, even more blood would be required. The aim of the present study was therefore to optimise the *lux* assay to use smaller volumes of blood and thereby increase its suitability for field studies in small children.

## 2 Materials and methods

### 2.1 BCG-*lux* assay

The original development of the BCG-*lux* assay has been described elsewhere in detail (Kampmann et al., 2000). In this study we made modifications to the volumes of blood used per assay, but not to the reporter-gene construct or the previously established multiplicities of infection and basic handling of the samples. Briefly, *M. bovis–BCG* transformed with a replicating vector containing the luciferase (lux) gene of *Vibrio harveyi* was prepared as previously described (Snewin et al., 1999). Frozen aliquots of BCG-*lux* bacilli were grown to midlog phase in Middlebrook 7H9 broth supplemented with 10% albumin dextrose catalase enrichment (BD; Franklin Lakes, NJ) and 15 μg/mL hygromycin (Roche, Lewes, UK). The bacilli were then diluted to a stock of 10^7^ Relative Light Units (RLU). This equates to an inoculum of about 10^6^ Colony Forming Units (CFU)/mL of blood.

Following informed consent, up to 10 mL of blood was collected from healthy adult volunteers into preservative-free heparin tubes (15 USP units sodium heparin/mL, BD Bioscience) and comparative assays with varying blood volumes were set up.

Blood was diluted 1:1 with RPMI 1640/2 mM glutamine/25 mM HEPES (N-2-hydoxyethylpiperazine-N′-ethane sulfonic acid) buffer (Sigma, Poole, UK) and infected with BCG-*lux* bacilli stock (1 × 10^7^ RLU) at a 1:10 concentration. This corresponded to a multiplicity of infection (mononuclear phagocyte to bacillus) of approximately 1:1, based on an established correlation of 10 RLU to 1 CFU. The infected diluted blood was then dispensed into triplicate aliquots of 1 mL, 0.67 mL and 0.5 mL for each time point (baseline t = 0 and t = 96h) and t = 96 samples were incubated at 37 °C on a rocking platform. Controls were set up in the same way using the same concentrations of mycobacteria in 7H9 culture medium.

At each time point the aliquots were processed as described below and supernatants were collected for future measurement of cytokine profiles. Aliquots were centrifuged for 10 min at 2000 g and supernatants were collected and stored at − 20 °C (300 μL for 1 mL aliquots, 200 μL for 0.67 mL aliquots and 150 μL for 0.5 mL aliquots). PBS was added in replacement for the withdrawal of supernatant and distilled water was added 1:10 to lyse the red blood cells followed by incubation for 10 min maximum at room temperature. The tubes were spun at 2000 g for 10 min and the supernatants removed. The pellet was resuspended in 1 mL PBS and a few glass beads were added to help disperse the pellet.

The samples were then measured in the luminometer (Berthold AutoLumat Plus, Berthold Technologies, Germany) by diluting 1:10 in PBS and using 1% N-decyl aldehyde (Sigma-Aldrich, US) as the substrate. Mycobacterial luminescence was measured at baseline and at 96 h, and the growth ratio was calculated by division of the mean 96-hour luminescence value by the mean baseline value.

The control samples were processed in the same way excluding the centrifugation step for removal of supernatant and lysis of red blood cells, as these are irrelevant for bacteria growing in growth medium alone. Growth ratios were calculated and compared between different volumes of growth media, equivalent to the equation used for whole blood assays.

### 2.2 Statistical evaluation

A mean of the triplicate growth ratios was calculated for each sample per blood/control volume. A cross sectional comparison of the median growth ratio for all data available at each volume was compared using the non-parametric Kruskal–Wallis test. Analysis including only samples with repeated measures for each volume was carried out using Friedmann ANOVA test. In addition separate comparisons between each volume were analysed using Mann Whitney paired non-parametric tests. In all cases p ≤ 0.05 was termed significant.

## 3 Results

**Fig. 1:**
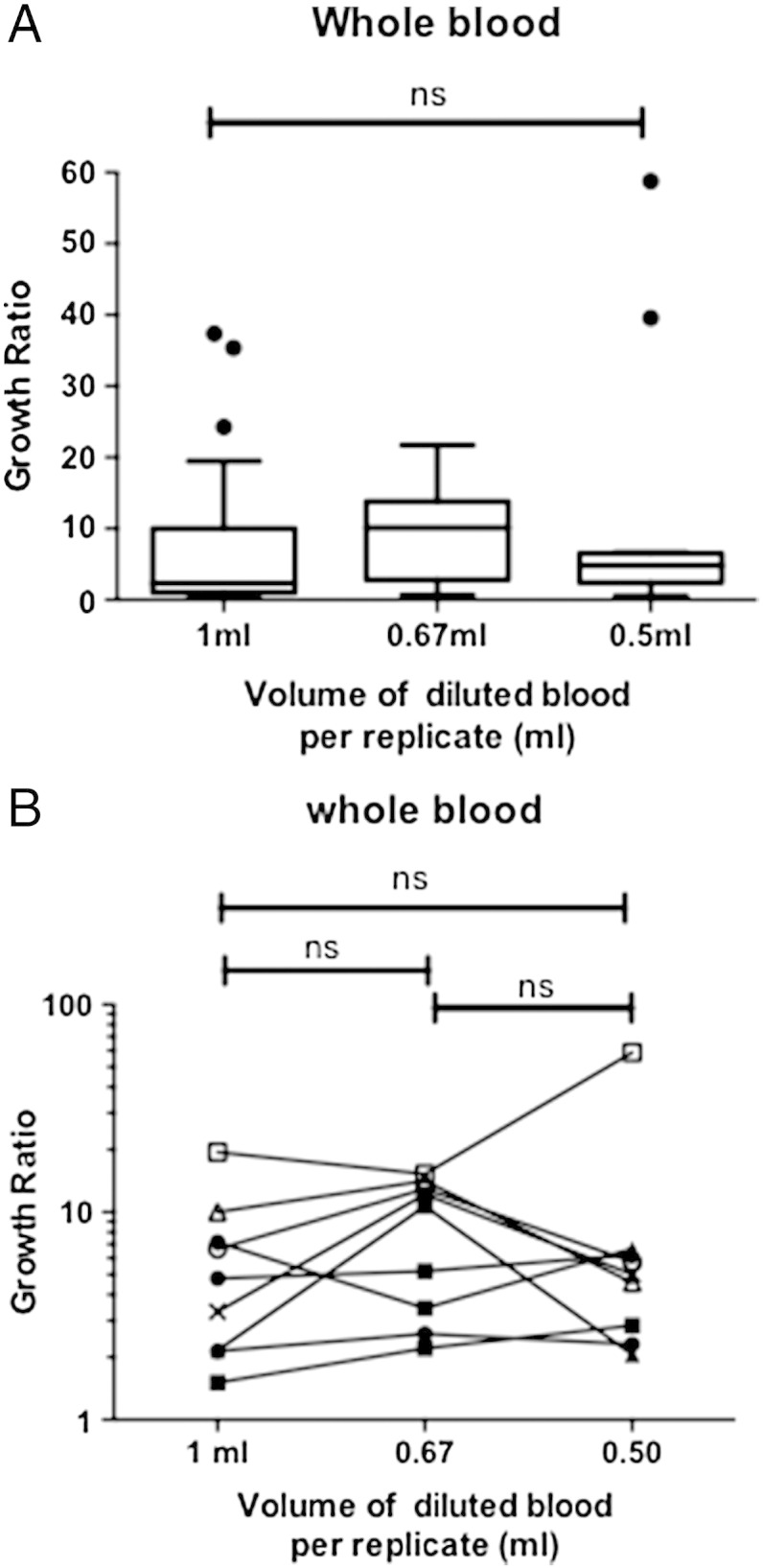
The BCG growth ratios for each volume of blood used in the BCG-*lux* assay. (A) Comparing all data for the different volumes of blood used using the Kruskal–Wallis test, n = 12–27 (B) Comparing data that included all three volumes tested using a Friedman ANOVA test and a paired non-parametric Mann Whitney test on comparisons of individual volumes. Each data point represents the average of three replicates from each donor in a given experiment, n = 9. For all tests p ≤ 0.05 was termed significant, ns = not significant.

Repeated results were obtained from 9 healthy adults. Initial studies that verified the methods were performed using the original volume of blood and therefore there are more values at the 1 mL volume than at the smaller volumes. These data points were included in the initial analysis. There was no significant difference between the median growth ratios for each of the volumes of diluted blood (Fig. 1A, p = 0.160) showing a 2-fold reduction in volume is possible in this assay system.

Examining the data that included samples that had all three different volume measurements, we found that there were no significant differences between any of the volumes (Fig. 1B, p = 0.398). Additional analysis comparing each volume of blood with the original volume of 1 mL also showed no significant differences between each of the volumes tested (Fig. 1B).

There were no significant differences between the different volumes of growth media for the growth of BCG (data not shown).

It was also established that using polystyrene round bottom tubes with snap caps (BD Biosciences) instead of 5 mL bijous for the incubation reduced the risk of disrupting the pellet while collecting the supernatants without any changes to the resulting mycobacterial growth (data not shown).

## 4 Conclusions

The aim of this study was to optimise the conditions of the BCG-*lux* assay to allow smaller volumes of blood to be used. Our results suggest that initial blood volumes as low as 250 μL per condition per replicate can provide the same data as the original 500 μL used and therefore a minimum of 2 mL of blood would be required for these assays instead of the currently used 4 mL.

A major limiting factor of studying infant immunity is the volume of blood that can be collected thereby reducing the number of assays or conditions possible within the study. Molecular assays have advanced in such a way that many parameters can be measured within one sample and has led to large scale genetic studies in infant populations, but they cannot measure growth restriction as a functional read-out. Immunological assays often require large numbers of cells from large volumes of blood and in the case of cell phenotyping, can be expensive. In this current lux assay, growth of mycobacteria is measured within whole blood samples reducing the need to manipulate the cells and thereby reducing the loss of cells in an already small volume of blood. The initial protocol required a minimum of 4 mL of blood and would therefore restrict any further assays being performed on the same sample, except that cytokines can be measured in the supernatants and RNA collected from the pellet, as previously described. We now show that this volume can be reduced to 2 mL with the same results.

We have previously demonstrated immunogenicity of BCG vaccine using this growth-restriction assay and established the assay as a useful tool for vaccine assessment and to decipher mechanisms of growth restriction. The ability to use reduced volumes of blood will further enhance its utility in trials of new tuberculosis vaccines in paediatric populations to assess how efficient a given novel vaccine may be against inhibiting mycobacterial growth *in vitro*. Since the most recent TB vaccine trial did not show protection despite predicted immunogenicity measured by cellular immune-assays (Tameris et al., in press), the addition of field friendly growth-inhibition assays in the next generation of vaccine trials is timely. We believe that the lux assay could play a role in such clinical trials.

## Role of the funding source

The study was supported by the funding from the Medical Research Council (UK) to BK and SB. Funders did not participate in the study design, collection, analysis and interpretation of data; in the writing of the report; and in the decision to submit the article for publication.
